# SAMHD1 acetylation enhances its deoxynucleotide triphosphohydrolase activity and promotes cancer cell proliferation

**DOI:** 10.18632/oncotarget.19704

**Published:** 2017-07-31

**Authors:** Eun Ji Lee, Ji Hae Seo, Ji-Hyeon Park, Tam Thuy Lu Vo, Sunho An, Sung-Jin Bae, Hoang Le, Hye Shin Lee, Hee-Jun Wee, Danbi Lee, Young-Hwa Chung, Jeong A. Kim, Myoung-Kuk Jang, Soo Hyung Ryu, Ensil Yu, Se Hwan Jang, Zee Yong Park, Kyu-Won Kim

**Affiliations:** ^1^ SNU-Harvard NeuroVascular Protection Research Center, College of Pharmacy and Research Institute of Pharmaceutical Sciences, Seoul National University, Seoul 08826, Korea; ^2^ Department of Biochemistry, Keimyung University School of Medicine, Daegu 42601, Korea; ^3^ Department of Internal Medicine, University of Ulsan College of Medicine, Asan Medical Center, Seoul 05505, Korea; ^4^ DNA Link Inc., Seoul 03759, Korea; ^5^ Department of Internal Medicine, Hallym University College of Medicine, Kangdong Sacred Heart Hospital, Seoul 05355, Korea; ^6^ Department of Internal Medicine, Inje University College of Medicine, Seoul Paik Hospital, Seoul 04551, Korea; ^7^ Department of Pathology, University of Ulsan College of Medicine, Asan Medical Center, Seoul 05505, Korea; ^8^ School of Life Sciences, Gwangju Institute of Science & Technology, Gwangju 61005, Korea; ^9^ Crop Biotechnology Institute, GreenBio Science and Technology, Seoul National University, Pyeongchang 25354, Korea

**Keywords:** SAMHD1, acetylation, dNTPase, cell cycle, cancer

## Abstract

SAM domain and HD domain containing protein 1 (SAMHD1) is a deoxynucleotide triphosphohydrolase (dNTPase) that inhibits retroviruses by depleting intracellular deoxynucleotide triphosphates (dNTPs) in non-cycling myeloid cells. Although SAMHD1 is expressed ubiquitously throughout the human body, the molecular mechanisms regulating its enzymatic activity and function in non-immune cells are relatively unexplored. Here, we demonstrate that the dNTPase activity of SAMHD1 is regulated by acetylation, which promotes cell cycle progression in cancer cells. SAMHD1 is acetylated at residue lysine 405 (K405) *in vitro* and *in vivo* by an acetylatransferase, arrest defective protein 1 (ARD1). Acetylated SAMHD1 wildtype proteins have enhanced dNTPase activity *in vitro*, whereas non-acetylated arginine substituted mutants (K405R) do not. K405R mutant expressing cancer cells have reduced G1/S transition and slower proliferation compared to wildtype. SAMHD1 acetylation levels are strongest during the G1 phase, indicating a role during G1 phase. Collectively, these findings suggest that SAMHD1 acetylation enhances its dNTPase activity and promotes cancer cell proliferation. Therefore, SAMHD1 acetylation may be a potent therapeutic target for cancer treatment.

## INTRODUCTION

Deoxyribonucleotide triphosphates (dNTPs) are the precursors of DNA synthesis, and their strict balance is critical for proper DNA replication and repair in cells [1]. When cells proliferate, the availability of dNTP fluctuate: the largest pools are seen during S phase and the smallest in G0, which controls the initiation of DNA replication [2]. This fluctuation needs to remain within a range optimal for chromosomal replication in order to maintain genomic stability; thus eukaryotes use diverse mechanisms to strictly regulate the supply of dNTPs [3]. Ribonucleotide reductases (RNR) are the major contributors that synthesize dNTPs from ribonucleoside diphosphates [4]. RNRs have long been recognized as an attractive target for cancer treatment, because their dysregulated activity is associated with genomic instability, malignant transformation and cancer development [5].

An additional mode of dNTP pool control was discovered in 2011 by the enzyme SAMHD1 (Sterile alpha motif domain and Histidine-aspartic domain containing protein 1). With its deoxynucleotide triphosphohydrolase (dNTPase) activity, SAMHD1 can cleave dNTPs, yielding deoxyribonucleosides plus tripolyphosphate [6, 7]. Originally discovered as a component of the human innate immune system [8], SAMHD1 depletes the level of cellular dNTPs to an amount that is insufficient for reverse transcription to block early-stage retroviral replication in dendritic and other myeloid cells, and, in particular, has anti-viral activity against HIV-1 [9, 10]. In addition, SAMHD1 stimulates cell proliferation in lung fibroblasts and THP-1 cells as its levels were variably expressed during cell cycle progression [11, 12]. Because SAMHD1 can control the availability of dNTPs and cell cycle progression, it is likely associated with cancer; however, little has been investigated about its role in cancer proliferation.

Posttranslational modifications (PTMs), such as phosphorylation, glycosylation, and acetylation, are chemical modifications of a protein that increase its functional diversity [13]. Phosphorylation of SAMHD1 has been reported to regulate its anti-viral restriction activity [14], but not its dNTPase activity [15]. Other PTMs or mechanisms that affects its dNTPase activity directly have not been discovered. Protein acetylation is one of the major PTMs in cell signaling and metabolism in eukaryotes as its regulation is crucial for important cellular processes [16–18]. Arrest defective protein 1 (ARD1, also known as Naa10) is an acetyltransferase that acetylates various substrates to alter their mode of action, including ARD1 itself (autoacetylation) [19], androgen receptor (AR) [20], Runt-related transcription factor 2 (Runx2) [21] and heat shock protein 70 (HSP70) [22]. Moreover, ARD1 is suggested to be oncogenic and overexpressed in several types of cancers, including breast [23, 24], prostate [20], lung and colorectal [25].

In the present study, we found that SAMHD1 is acetylated by ARD1. As ARD1 is reported to be oncogenic and the enzymatic activity of SAMHD1 is relevant to the cell cycle regulation, we hypothesized that ARD1-mediated SAMHD1 acetylation may contribute to cancer cell proliferation. By identifying the specific acetylation site of SAMHD1 and mutating it to block its acetylation, we aimed to discover a novel function of SAMHD1 acetylation in cancer cell growth.

## RESULTS

### SAMHD1 is a novel acetylation substrate of ARD1

**Figure 1:**
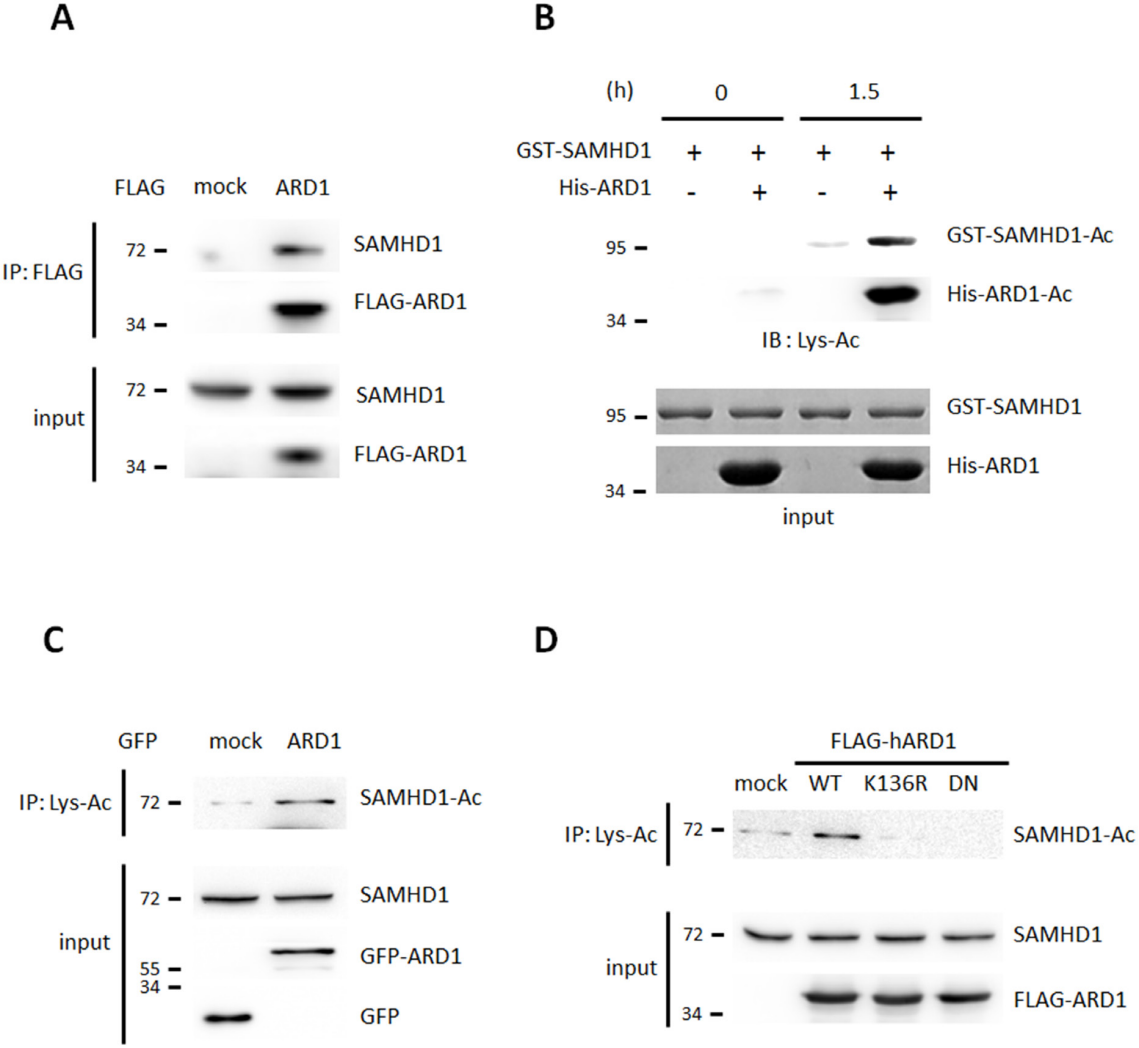
SAMHD1 is a novel acetylation substrate of ARD1 **(A)** FLAG-ARD1 was immunoprecipitated from HEK293T cells, and co-precipitation of endogenous SAMHD1 was analyzed by western blot. **(B)** Recombinant GST-SAMHD1 proteins were subjected to *in vitro* acetylation assay with ±His-ARD1. The acetylation level was determined with anti-Lys-Ac antibody. **(C)** Lys-acetylated proteins were precipitated from GFP-mock or ARD1 expressing HEK293T cells and were blotted with anti-SAMHD1 antibody. **(D)** FLAG-ARD1 wildtype, K136R and DN mutants were overexpressed in HEK293T. Lysed proteins were precipitated with anti-Lys-Ac antibody and were blotted with anti-SAMHD1 antibody.

While screening for ARD1-binding proteins using affinity purification combined with mass spectrometry, we found that endogenous SAMHD1 binds to ARD1 in HEK293T cells (Supplementary Figure 1A). This was confirmed by co-immunoprecipitation followed by western blot analysis (Figure 1A). As ARD1 is an acetyltransferase, we next tested if ARD1 acetylates SAMHD1 directly. An *in vitro* acetylation assay revealed that recombinant GST-SAMHD1 protein was acetylated when His-ARD1 and acetyl-CoA were present (Figure 1B). The intensity of endogenous acetylated SAMHD1 was stronger in GFP-ARD1-overexpressing HEK293T cells, indicating that ARD1-mediated acetylation also occurs *in vivo* (Figure 1C). ARD1 is autoacetylated at its K136 residue to enhance its catalytic activity [19]. To test whether this is required for SAMHD1 acetylation, we overexpressed K136R and dominant negative (DN) ARD1 mutants to inhibit this activity. These two variants blocked SAMHD1 acetylation (Figure 1D). This data demonstrates that SAMHD1 is acetylated by autoacetylated-ARD1, which is required as an up-stream signal.

### SAMHD1 is acetylated at K405 by ARD1

**Figure 2:**
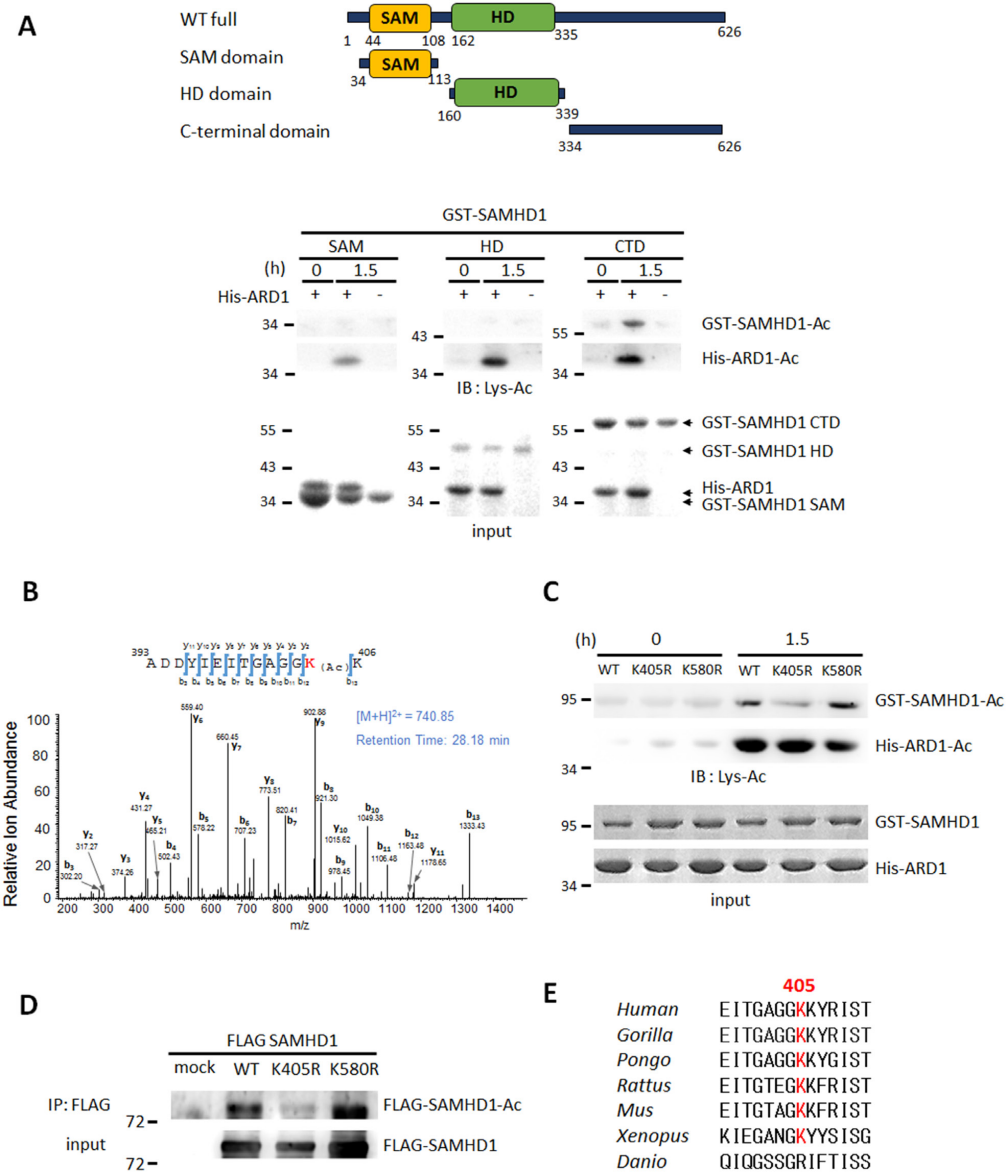
SAMHD1 is acetylated at K405 by ARD1 **(A)** Construction of SAMHD1 deletion mutants (left). SAM; SAM domain, HD; HD domain, CTD; C-terminal domain. Deletion mutants of GST-SAMHD1 were subjected to *in vitro* acetylation assays with ±His-ARD1 and blotted with anti-Lys-Ac antibody (right). Arrows indicate respective proteins. **(B)** GST-CTD were acetylated *in vitro* and subjected to LC–MS/MS. **(C)** GST-SAMHD1 recombinants were subjected to *in vitro* acetylation assays with His-ARD1. Acetylation levels were determined with an anti-Lys-Ac antibody. **(D)** FLAG-SAMHD1 variants were immnunoprecipitated from HEK293T cells, and their acetylation levels *in vivo* were analyzed using an anti-Lys-Ac antibody. **(E)** Sequence alignment of SAMHD1 K405 residue in various species.

Next, to identify the target site of acetylation, we constructed deletion mutants of SAMHD1 and subjected them to an *in vitro* acetylation assay in the presence of His-ARD1 and acetyl-CoA. Among 3 constructs, C-terminal domain (CTD) was found to be the major acetylated domain (Figure 2A). To determine which residue in CTD is acetylated by ARD1, acetylated GST-CTD was digested into peptides, and subjected to micro-liquid chromatography-tandem mass spectrometry (LC–MS/MS). 2 potential acetylation sites were revealed, K405 and K580 (Figure 2B, Supplementary Figure 1B). To establish the causality of this relationship and to verify the critical site between the two, we generated K405R and K580R mutations, in which the lysine residues were replaced by arginine. When subjected to *in vitro* acetylation in the presence of ARD1, only K405R had decreased acetylation, indicating that K405 is the main target site for acetylation (Figure 2C). This result was also observed *in vivo* as K405R SAMHD1 had decreased acetylation in HEK293T cells compared to wildtype or K580R-expressing cells (Figure 2D). Alignment of SAMHD1 amino acid sequence from various species revealed that the K405 residue is well-conserved from human to *Xenopus*, suggesting that this acetylation site may be critical for its biological functions in many species (Figure 2E). In summary, ARD1-mediated SAMHD1 acetylation occurs at the conserved K405 residue.

### SAMHD1 acetylation upregulates its dNTPase activity

**Figure 3:**
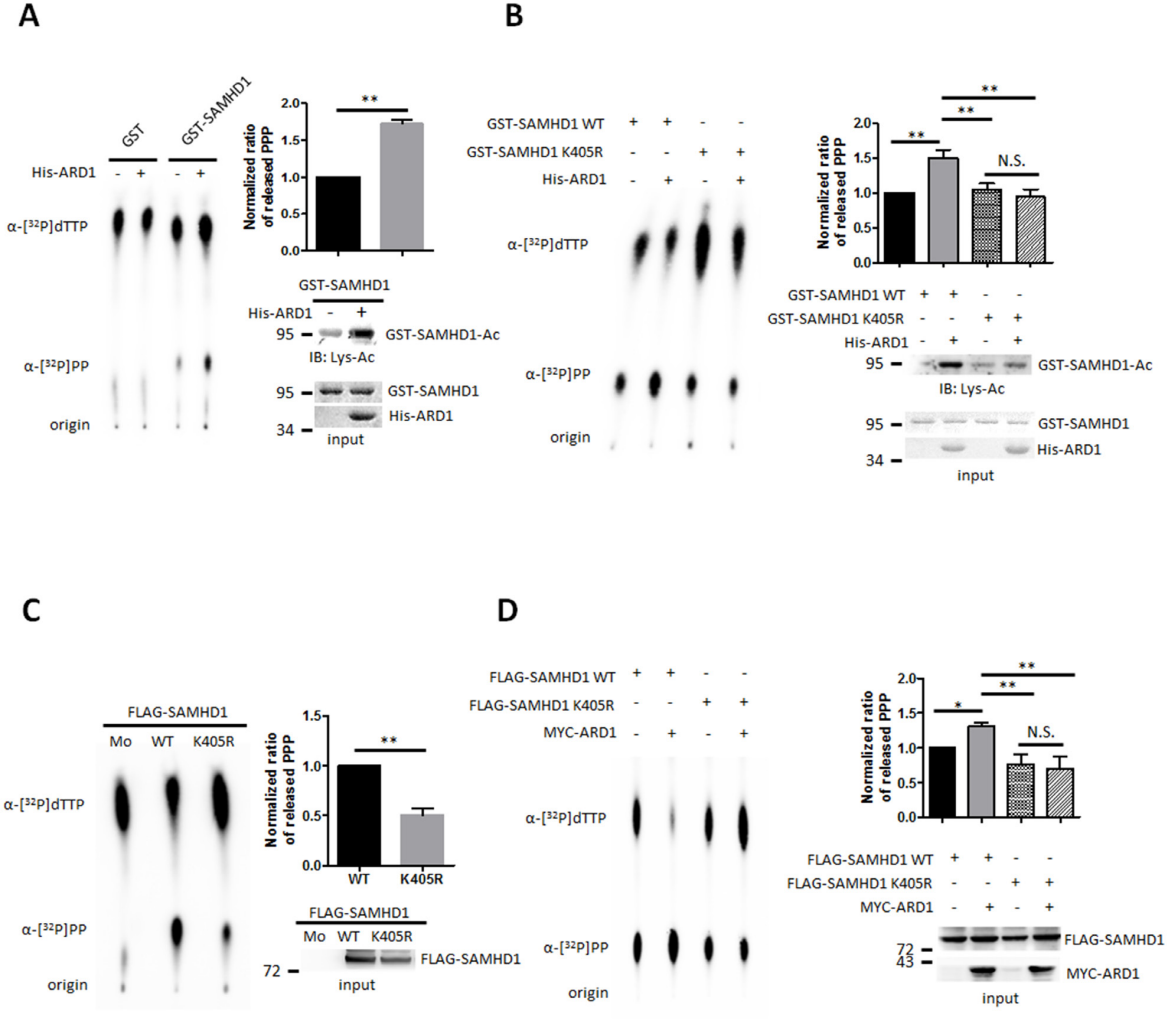
SAMHD1 acetylation upregulates its dNTPase activity **(A** and **B)** Recombinant GST-SAMHD1 proteins were acetylated *in vitro* with ±His-ARD1 and blotted with anti-Lys-Ac antibody (lower right). Products were subjected to dGTP-triphosphohydrolase assay and imaged using a phosphorimager (left). The released amount of α-[^32^P]PP were measured and presented as mean ±S.D. (n=3) (upper right). **(C** and **D)** FLAG-mock, SAMHD1 wildtype or K405R mutants were overexpressed with ±MYC-ARD1, then FLAG-tagged proteins were immunoprecipitated from HEK293T (lower right). Precipitated proteins were subjected to dGTP-triphosphohydrolase assay (left) and were quantified (upper right). Shown are mean ±S.D. (n=3). *P<0.05; **P<0.01; *t* test, N.S.; not significant.

We next investigated whether SAMHD1 acetylation could regulate its dNTPase activity directly. We tested the ability of recombinant acetylated and non-acetylated SAMHD1 to hydrolyze α-^32^P labeled thymidine triphosphate (α-[^32^P]TTP) to deoxythymidine (dT) and α-[^32^P]PP. Acetylated and non-acetylated GST-SAMHD1 proteins were incubated with α-[^32^P]TTP, then were separated using thin layer chromatography to compare the amount of released α-[^32^P]PP. Acetylated SAMHD1 had higher enzymatic activity over the non-acetylated protein (Figure 3A), and this effect was lost in the non-acetylated K405R mutant (Figure 3B). In accordance, wildtype SAMHD1 immunoprecipitated from HEK293T cells also had enhanced enzymatic activity over the K405R mutant (Figure 3C). When co-expressed with MYC-ARD1, the amount of hydrolyzed α-[^32^P]PP increased in SAMHD1 wildtype expressing cells, but not in K405R mutant cells (Figure 3D). These results suggest that the ARD1-mediated SAMHD1 acetylation enhances its dNTPase activity.

### SAMHD1 acetylation is important for cell proliferation in various cancer cells

**Figure 4:**
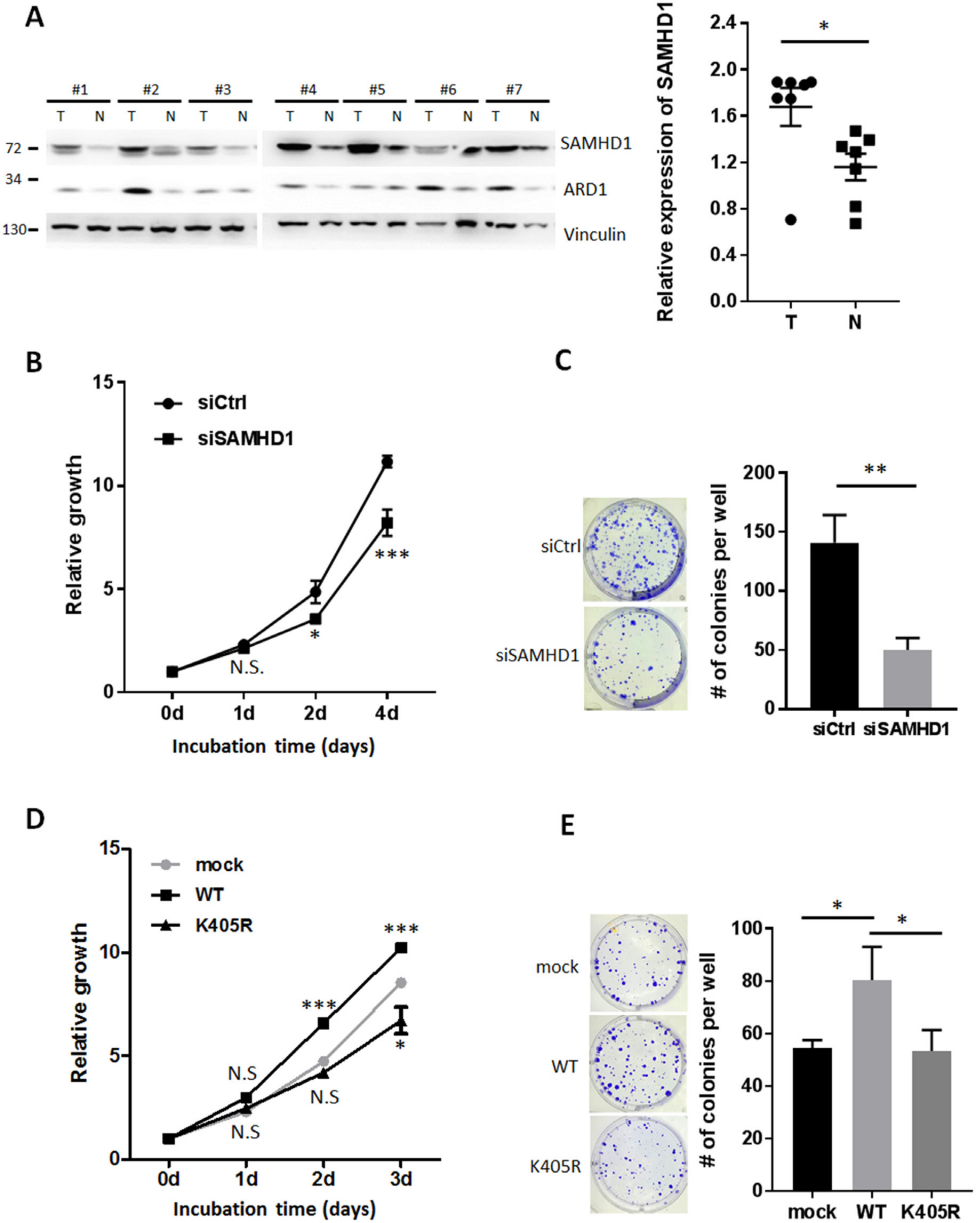
SAMHD1 acetylation is important for cell proliferation in various cancer cells **(A)** Tumor and non-tumor tissues from 7 hepatocarcinoma patients were lysed and extracted. Protein expression levels were analyzed by western blot and presented as mean ±S.E.M. (n=7). T, tumor tissue; N, non-tumor tissue. **(B)** Cell growth of siCtrl or siSAMHD1 treated HeLa cells were monitored over time by MTS assay. Shown are mean ±S.E.M. (n=4). **(C)** siCtrl or siSAMHD1 treated MCF7 cells were subjected to anchorage-dependent colony formation assays. Number of colonies were presented as mean ±S.D. (n=3) with representative well pictures. **(D)** Cell growth of stable HeLa cells expressing SAMHD1 wildtype, K405R or empty vector were monitored over time by MTS assay. Shown are mean ±S.E.M. (n=4). **(E)** Stable MCF7 cells expressing SAMHD1 wildtype, K405R or empty vector were subjected to anchorage-dependent colony formation assays. Number of colonies were presented as mean ±S.D. (n=3) with representative well pictures.*P<0.05; **P<0.01; ***P<0.001; *t* test; N.S.; not significant.

In proliferating cell populations, the supply of dNTPs needs to be strictly balanced for proper DNA replication and repair. As SAMHD1 controls the intracellular pool of dNTPs via its dNTPase activity, we hypothesized that SAMHD1 may also regulate cell proliferation in cancer. SAMHD1 expression levels in tumor and surrounding non-tumor tissues collected from 7 hepatocarcinoma patients were analyzed by western blot. The tumor tissues had higher levels of SAMHD1 than non-tumor tissues, suggesting that SAMHD1 may be related to hepatocellular tumorigenesis (Figure 4A). The level of ARD1 was also elevated in tumor tissues as previously reported [26], implying that ARD1-mediated SAMHD1 acetylation levels may be upregulated in hepatocarcinoma. To investigate the role of SAMHD1 in cancer cells, SAMHD1 was silenced in various cancer cell lines (Supplementary Figure 2A). SAMHD1-downregulation caused HeLa cells to grow slower than the control cells (Figure 4B), and caused a decrease in MCF7 colony formation (Figure 4C), indicating that SAMHD1 expression is important for cancer cell growth.

To determine whether SAMHD1 acetylation affects this phenomenon, we constructed stable cancer cell lines expressing wildtype SAMHD1 and K405R mutant (Supplementary Figure 2B). Unlike the K405R mutant, overexpression of wildtype SAMHD1 resulted in an increased growth rate in HeLa cells (Figure 4D). Moreover, only wildtype-expressing cells exhibited an increased number of colonies when subjected to the anchorage dependent/independent colony formation assay (Figure 4E, Supplementary Figure 2C). These results demonstrate that SAMHD1 acetylation enhances its ability to promote cancer cell proliferation.

### SAMHD1 acetylation promotes G1/S transition in cancer cells

**Figure 5:**
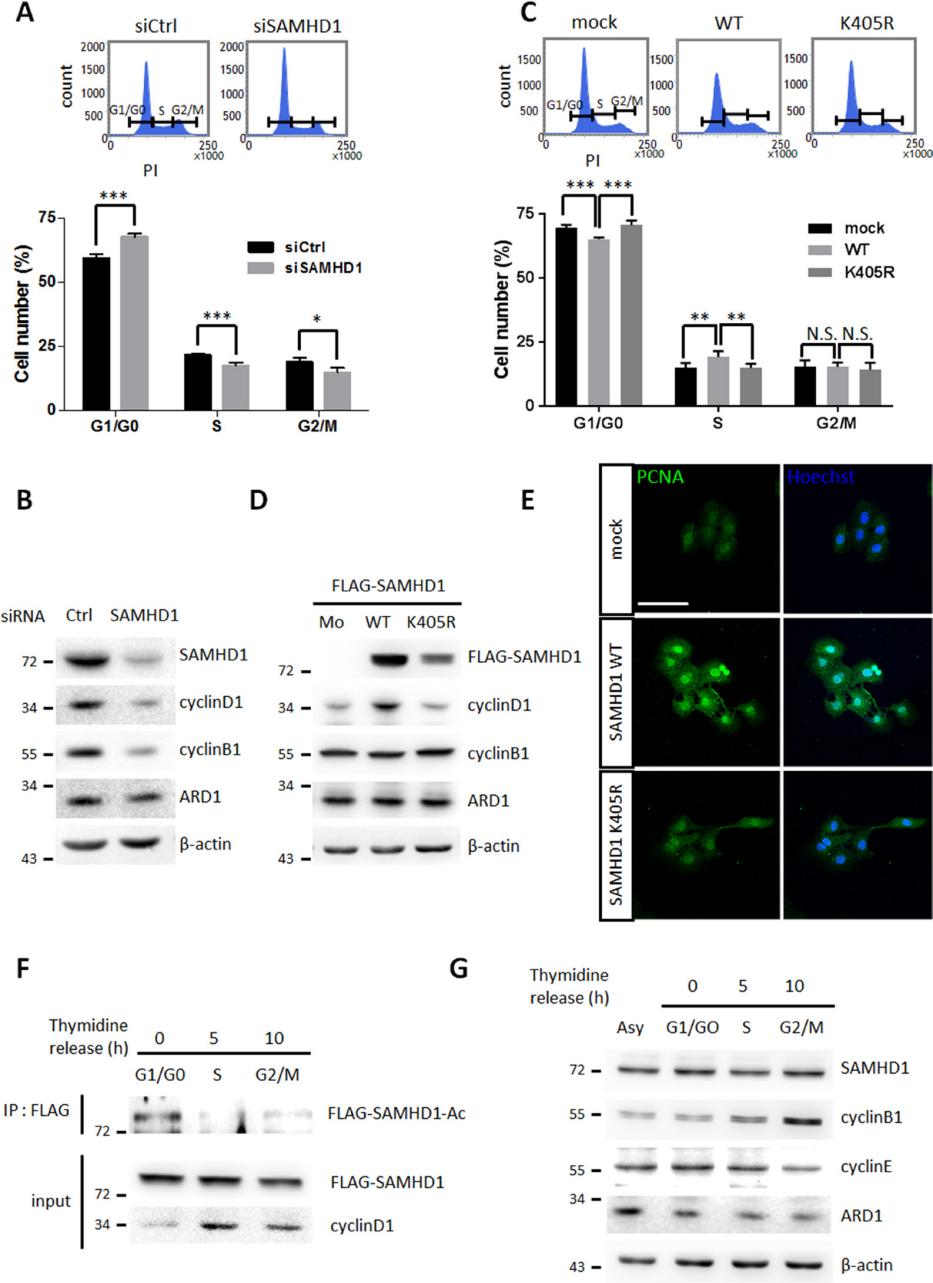
SAMHD1 acetylation promotes G1/G0 transition in cancer cells **(A)** Cell cycle profiles of siCtrl or siSAMHD1 treated HeLa cells were determined by flow cytometry. Representative cell cycle profiles (top); the percentage of cells in each phase is presented as mean ±S.D. (n=3) (bottom). **(B)** Lysates were extracted from siCtrl or siSAMHD1 treated HeLa cells and the levels of cyclin D1 and cyclin B1 were immunoblotted by corresponding antibodies. **(C)** Cell cycle profiles of stable A549 cells expressing SAMHD1 wildtype, K405R or empty vector were determined by flow cytometry. Representative cell cycle profiles (top); the percentage of cells in each phase is presented as mean ±S.D. (n=3) (bottom). **(D)** The levels of cyclin D1 and cyclin B1 were immunoblotted in the lysates extracted from stable A549 cells expressing SAMHD1 wildtype, K405R or empty vector. **(E)** Stable Hep3B cells expressing SAMHD1 wildtype, K405R or empty vector were serum-deprived for 48 h and were re-stimulated for 24 h, then the expressions of PCNA were monitored by confocal microscopy with immunofluorescence using the PCNA antibody. Hoechst was used for nuclei staining. Scale bar; 100 μm. **(F)** FLAG-SAMHD1 wildtype expressing stable A549 cells were synchronized to specific cell cycle phases using thymidine-double block method. SAMHD1 was precipitated and blotted with an anti-Lys-Ac antibody. **(G)** HeLa cells were synchronized to specific cell cycle phases by thymidine double block method. The expression levels of SAMHD1 was assessed by western blotting. *P<0.05; **P<0.01; ***P<0.001; *t* test; N.S.; not significant; Asy; asynchonized.

SAMHD1 has been previously reported to control the mammalian cell cycle by regulating dNTP availability [11, 12]. To determine whether cell cycle progression is altered by SAMHD1 in cancer cells, HeLa cells were treated with siSAMHD1 to downregulate the expression of SAMHD1. 48 h after treatment, SAMHD1-silenced HeLa cells were synchronized by 48 h of serum-starvation and re-stimulated for 24 h, then subjected to flow cytometry for cell cycle analysis. Depletion of SAMHD1 caused delay in G1 phase, as G1/G0 population increased whereas S and G2/M populations decreased (Figure 5A). Western blot analysis also revealed that the expression levels of cyclin D1 and B1—markers for G1/S and G2/M transition respectively—were decreased in siSAMHD1 treated cells (Figure 5B). Accordingly, in wildtype SAMHD1-expressing A549 cells but not K405R-expressing cells, the G1-to-S phase progression was increased (Figure 5C). This result was also confirmed by the expression levels of cyclin D1 (Figure 5D). Immunofluorescent images of proliferating cell nuclear antigen (PCNA)—mainly expressed during the S phase—also highlighted the increased S phase in wildtype SAMHD1 expressing cells (Figure 5E). Collectively, these results demonstrate that SAMHD1 acetylation contributes to G1/S transition in cancer cell lines.

To validate the role of SAMHD1 acetylation in cell cycle progression in detail, we measured acetylation levels during each phases. The G1/G0 phase had the greatest amount of acetylated SAMHD1 in A549 and HeLa cells (Figure 5F, Supplementary Figure 3A), suggesting that the dNTPase activity of SAMHD1 may be strongest during the G1/G0 phase. SAMHD1 expression levels are known to fluctuate throughout the cell cycle in fibroblasts [11]; however, there were no significant differences in Hela cells and Hep3B cells (Figure 5G, Supplementary Figure 3B), indicating that the altered cell cycle observed in wildtype SAMHD1- and mutant-expressing cells is caused by its acetylation and not changes in expression. In Summary, SAMHD1 acetylation promotes tumor growth by facilitating G1/S cell cycle progression.

## DISCUSSION

**Figure 6:**
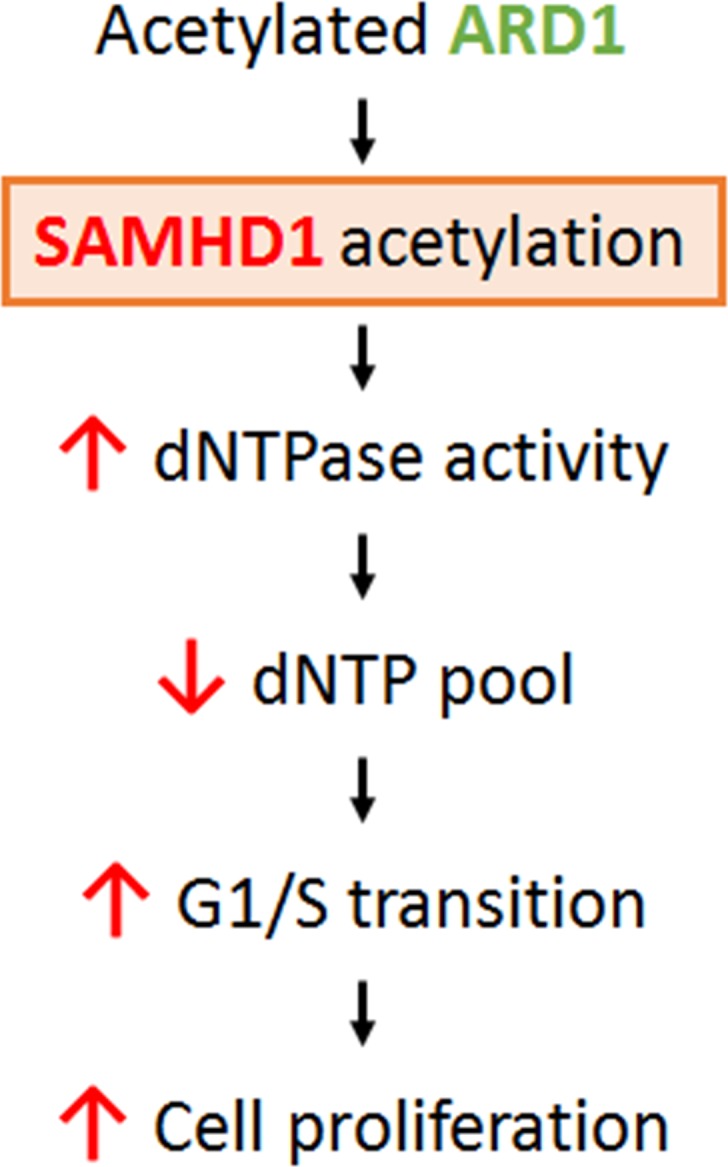
Schematic for the mechanism of ARD1-mediated SAMHD1 acetylation in cancer cell proliferation ARD1-mediated SAMHD1 acetylation facilitates G1/S transition by lowering dNTP levels and promotes cancer cell proliferation.

SAMHD1 was identified for its anti-HIV-1 activity; thus, most studies have focused on its antiviral function. However, SAMHD1 is expressed not only in immune cells but also ubiquitously in human organs, suggesting a regulatory mechanism for its enzymatic activity and additional functions in non-immune cells. Here, we identified SAMHD1 acetylation, a novel PTM for this protein, and its roles in cancer cell proliferation (Figure 6). The K405R residue of SAMHD1 is acetylated by ARD1, resulting in increased dNTPase activity *in vitro*. SAMHD1 acetylation levels are highest during G1 phase and facilitates G1/S transition, causing increases in cell growth and colony formation in cancer cells. However, the K405R acetylation blocked-mutant with decreased dNTPase activity did not have the same effect, suggesting that SAMHD1 acetylation promotes the G1/S transition through its dNTPase activity. dNTP levels are known to affect cell cycle progression; for example, constitutively high dNTP concentration delays entry into S phase [2]. SAMHD1 is reported to promote S phase progression by sufficiently lowering cellular dNTP availability to satisfy G1 checkpoint [11, 12]. Thus, the increased level of SAMHD1 acetylation during G1 phase may cause sufficiently low levels of dNTPs for G1/S transition, which in turn promotes cancer cell proliferation (Figure 6). These findings broaden the understating of dNTP metabolism and cell cycle regulation, and highlights SAMHD1 and its acetylation as a novel therapeutic target for cancer treatment.

Along with K405 residue, LC-MS/MS analysis of acetylated CTD also revealed K580 residue as an acetylated site (Figure 2B, Supplementary Figure 1B). When tested *in vitro*, however, K580 acetylation was not affected by ARD1 (Figure 2C), and K580R mutation had no effect on its dNTPase activity (data not shown), indicating that K580 acetylation may not play a meaningful role in ARD1-mediated SAMHD1 activation. However, we will not rule out the possibility that K580 residue-acetylation may carry out other functions of SAMHD1.

SAMHD1 acetylation may also affect its anti-viral activity in non-cycling immune cells, because the ability of SAMHD1 to deplete dNTP pools is critical for limiting lentiviral replication in non-cycling immune cells [6, 15, 27]. The sole expression of SAMHD1 itself was insufficient to induce this effect in proliferating immune cells, implicating the existence of PTMs and/or a cofactor expressed only in non-proliferating cells [28, 29]. Here, we have discovered that SAMHD1 acetylation enhances its dNTPase function directly as acetylated SAMHD1 had higher hydrolase activity over non-acetylated proteins and the K405R mutants (Figure 3). Moreover, previous report has demonstrated that histone deacetylase inhibitors (HDACi) possess antiretroviral activity that is SAMHD1–dependent in macrophages; which suggests that protein acetylation related to SAMHD1 plays role in viral defense [30]. Therefore, further investigations on acetylation status in cycling/non-cycling myeloid cells are needed for a deeper understanding of the role of SAMHD1 acetylation in immune defense.

## MATERIALS AND METHODS

### Human liver tissue samples

Hepatocarcinoma tissues and surrounding non-tumorous liver samples were obtained from patients at Asan Hospital (Seoul, Republic of Korea). Clinicopathological characteristics of hepatocarcinoma patients are summarized in Supplementary Table 1. This study was approved by Institutional Review Board of Asan Hospital, and written informed consent was obtained from all the patients.

### Cell culture and synchronization

HeLa, A549, Hep3B, MCF-7 and HEK293T cells were purchased from ATCC. Cells were grown in DMEM supplemented with 10% fetal bovine serum (FBS) and 1% penicillin/streptomycin in 5% CO_2_ humidified atmosphere at 37°C. For serum starvation, cells were incubated in DMEM without serum for 48 h and re-stimulated with DMEM containing 10% FBS. A double thymidine block was performed to synchronize cell populations in S phase. Cells were treated with 2 mM thymidine (Sigma-Aldrich) for 18 h, cultured for 9 h in normal growth medium, and then retreated with thymidine for another 16 h. After removing the thymidine, cells were released to normal medium and harvested at the indicated times.

### *in vitro* acetylation assay

Acetylation assay was performed as described previously [19]. Briefly, 1 μg of purified recombinants or precipitated cellular proteins were incubated in total 20 μl of reaction mixture containing 50 mM Tris–HCl (pH 8), 0.1 mM EDTA, 1 mM DTT, 10% glycerol and 10 mM acetyl-CoA at 37°C for 1.5 h. Reaction products were separated by SDS–PAGE and were analyzed by western blot using anti-Lys-Ac antibody. Input proteins were visually quantified using Coomassie brilliant blue staining or Ponceau S staining.

### Mass spectrometric analysis

Mass spectrometric analysis was performed as previously described [31]. For enzymatic digestion of recombinant SAMHD1, two GST-SAMHD1 samples used in the *in vitro* acetylation assay (incubated with ±ARD1) were immediately denatured using 6 M GuHCl and were reduced with 5 mM DTT for 30 min at room temperature, then alkylated with 25 mM iodoacetamide for 30 min. Glu-C digestions were carried out for 6 h at 25°C then were quenched by 5% formic acid. Micro RPLC–MS/MS analysis was performed as previously described [22].

### dGTP-triphosphohydrolase assay

An enzymatic assay based on thin-layer chromatography was performed as described previously [9]. The purified recombinant or immunoprecipated cellular protein (1 μg) was incubated in total 20 μl of reaction mixture composed of 50 mM Tris-Cl (pH 8.0), 20 mM KCl, 5 mM MgCl_2_, 0.1 μCi [α-^32^P]dTTP, and 200 μM ice-cold dGTP for 3 h at 37°C. The reactions were stopped by heat inactivation at 70°C for 10 min. Mixtures were blotted onto polyethyleneimine (PEI)-cellulose plates (Sigma-Aldrich) and subsequently separated using a mobile phase of 1.2 M LiCl. After separation, the α-^32^P-labeled reaction products were visualized using a phosphorimager. Results from 3 independently-repeated experiments were analyzed.

### siRNA and plasmid construction

The siRNA sequence targeting human SAMHD1 corresponds to GAUUCAUUGUGGCCAUAUA as previously described [9]. Full-length of cDNAs for human SAMHD1 (Genbank: NM_015474) and human ARD1 (Genbank: NM_003491.3) were obtained from PCR and subcloned into pCDNA3.1 (FLAG-SAMHD1), pCMV-tag2b (FLAG-ARD1), pEGFP-C3 (GFP-ARD1) and pCS2^+^ (MYC-ARD1) vectors for cellular expression or pGEX-4T (GST-SAMHD1) and pET28a (His-ARD1) vectors for the bacterial induction of recombinant proteins. Deletion mutants of GST-SAMHD1 were constructed from pGEX-4T-SAMHD1 plasmid. cDNAs of SAMHD1 corresponding to 34–113 aa, 160–339 aa and 334–626 aa were amplified by PCR and inserted into pGEX-4T-1. For construction of stable cell lines, cDNAs of SAMHD1 was co-inserted into pMX-IRES-Blasticidin^R^ vector with PCR-amplified FLAG. Point mutations in SAMHD1 (K405R and K580R) and ARD1 (K136R and DN: R82A/Y122F) were generated using the Muta-Direct Site Directed Mutagenesis kit (Intron) according to the manufacturer's instructions. The following primers and its reverse-complement for each point mutation (mutated based in lower case bold):

SAMHD1 K405R: TACAGGTGCTGGAGG**cgg**AAAGTATCGCATTTC

SAMHD1 K580R: GACAGAAATTTCACC**cg**GCCGCAGGATGGCGAT

ARD1 K136R: AGTGAAGTGGAGCCCA**g**ATACTATGCAGATGGG

ARD1 R82A: TGTGAAGCGTTCCCAC**gc**GCGCCTCGGTCTGGCT

ARD1 Y122F: GCCGCCCTGCACCTCT**t**TTCCAACACCCTCAAC

### Transfection and stable cell line construction

Transfection was performed as described previously [22]. We transfected HEK293T cell with polyethyleneimine (PEI) at a ratio of 3:1 (μl PEI/μg plasmid DAN) in basal media overnight. siRNA targeting SAMHD1 was transfected with HiPerFect reagent (Quiagen) according to the manufacture's instruction.

For the establishment of stable cells, pIRES-FLAG-SAMHD1 plasmids were transfected into Platinum A cells and retroviral supernatants were harvested 72 h after transfection. Collected supernatants were used to transduce target cell lines (HeLa, A549, Hep3B and MCF-7) with polybrene (10 μg ml^−1^). Transduced cells were selected using blasticidin (10 μg ml^−1^). Expression of FLAG-SAMHD1 was quantified by western blot.

### Immunoblotting and immunoprecipitation

Cellular proteins were extracted using cell lysis buffer composed of 20 mM Tris-CL (pH 7.5), 150 mM NaCl, 0.1 mM EDTA, 0.1% Triton X-100 and a protease inhibitor cocktail (Roche). 20 ∼50 μg of cell extracts were used for immunoblotting. For immunoprecipitation, 1 mg of proteins were incubated with corresponding primary antibody conjugated to A or G bead (Upstate) or Anti-DDDDK-tag mAb-Magnetic Agarose (MBL) overnight at 4°C. Beads were washed three times with washing buffer containing 20 mM Tris-Cl (pH 7.5), 150 mM NaCl and 0.1 mM EDTA. After SDS–PAGE, membranes were immunoblotted using the corresponding primary antibody overnight at 4°C. HRP-conjugated secondary antibodies were incubated with the membranes for 1 h at room temperature. Visualization was performed using ECL Plus (Intron) and LAS-4000 (GE Healthcare).

### Screening of binding proteins

The protein-protein interaction assay was performed as reported previously [22]. HEK293T cells were transiently transfected with FLAG-HA tagged ARD1 and were lysed in lysis buffer consisting 20 mM Tris-Cl (pH 7.5), 150 mM NaCl, 0.1 mM EDTA, 0.2% Triton X-100 and a protease inhibitor cocktail (Roche). Cell lysates were incubated with anti-M2 resin (Sigma) for 2 h at 4°C, then resins were collected by centrifugation and washed three times with washing buffer, composed of 20 mM Tris-Cl (pH 7.5), 150 mM NaCl and 0.1 mM EDTA. Bound proteins were eluted by 3 × FLAG-peptide and were immunoprecipitated again using anti-HA-antibody for 2 h at 4°C. The bound proteins were analyzed by SDS–PAGE and silver staining.

### Recombinant protein preparation

BL21 cells were transformed with plasmids pGEX-4T-1-SAMHD1 or pET28a-ARD1 and were grown to and OD600 of 0.6–0.8. 1 mM IPTG was added to induce GST-tagged SAMHD1 or His-tagged ARD1, then cells were grown overnight at 20°C. Cells were collected and proteins were extracted with a lysis buffer consisting 50 mM Tris–HCl (pH 8), 250 mM NaCl, 2.5 mM EDTA and 1% Triton X-100. GST-tagged and His-tagged proteins were purified with Glutathione Sepharose 4B (GE Healthcare) and TALON^®^ Metal Affinity Resins (Clontech), respectively. Resin-bound proteins were eluted with an elution buffer containing 50 mM Tris–HCl (pH 8) and 10 mM reduced glutathione (GSH).

### Protein sequence alignment

SAMHD1 multiple protein sequence alignment was conducted by Constraint-based Multiple Alignment Tool provided from NCBI.

### Flow cytometry analysis

For a flow cytometry assay, cells were collected, fixed in 1% PFA, and stored at 4°C for 30 min. Cells were then washed with PBS and stained with propidium iodide (PI, 30 μg ml^−1^) mixed with RNase A (1 μg ml^−1^) for 15 min at 37°C. The DNA content of cells was assessed with a FACS-Verse system (BD Biosciences), and cell cycle profiles were analyzed with the BD FACSuite software. At least 30,000 cells in each sample were analyzed to obtain a measurable signal, using the same instrument setting. Results from 3 independently-repeated experiments were analyzed.

### Cell proliferation assay

The proliferation rates were measured using a Cell Proliferation Assay kit (Promega) following the manufacturer's instructions. Briefly, 2×10^3^ cells/well were seeded onto 96-well plates and were allowed to grow. At indicated time points, 20 μL of substrate solution was added to the cells and were incubated for 1 h for color development. The absorbance at 492 nm was measured to indicate the number of proliferating cells. Results from 4 replicates in one experiment were analyzed.

### Colony formation assay

Colony formation assays were performed as described previously [32]. For anchorage dependent colony formation, cells were seeded at a density of 100 cells/well onto 6-well plates and were allowed to grow for 2 weeks. Cells were then fixed with 100% methanol, stained with 0.005% crystal violet and the number of colonies were counted. For anchorage independent colony formation, cells were resuspended in 0.5 mL DMEM containing 0.4% agar and were seeded onto a layer of 1% agar in 12-well plates. After 2 weeks of incubation, colonies were stained with 0.005% crystal violet and counted. Results from 3 independently-repeated experiments were analyzed.

### Fluorescence microscopy

For the analysis of PCNA by fluorescence microscopy, cells were seeded onto the glass coverslips in 24-well plates. After synchronization, cells were fixed in 2% PFA for 20 min and were permeabilized in 1% triton X-100 in PBS 10 min at room temperature. Then, cells were incubated with PCNA antibody (Cell signaling) and visualized with Alexa 488-conjugated igG (Molecular Probes). Nucleus staining was performed with Hoechst 33342 (Molecular Probes). The immunofluorescence was visualized using a confocal microscopy (Carl Zeiss, LSM700).

### Statistical analysis

Results are expressed as the mean’s±s.d's or±s.e.m’s. *P* values were calculated by applying the two-tailed Student's *t* test or one-way ANOVA. A difference was considered statistically significant at a value of *P*<0.05.

## SUPPLEMENTARY MATERIALS FIGURES AND TABLE



## Abbreviations

SAMHD1: SAM domain and HD domain containing protein 1
dNTPase: deoxynucleotide triphosphohydrolase
dNTP: deoxynucleotide triphosphates
ARD1: arrest defective protein1
RNR: ribonucleotide reductase
HIV-1: human immunodeficiency virus 1
PTM: posttranslational modification
TTP: thymidine triphosphate
PCNA: proliferating cell nuclear antigen
IB: immunoblotting
IP: immunoprecipitation
Lys-Ac: lysine-acetylation

## References

[1] Reichard P (1988). Interactions between deoxyribonucleotide and DNA synthesis. Annu Rev Biochem.

[2] Chabes A, Stillman B (2007). Constitutively high dNTP concentration inhibits cell cycle progression and the DNA damage checkpoint in yeast Saccharomyces cerevisiae. Proc Natl Acad Sci U S A.

[3] Pai CC, Kearsey SE (2017). A critical balance: dNTPs and the maintenance of genome stability. Genes (Basel).

[4] Nordlund P, Reichard P (2006). Ribonucleotide reductases. Annu Rev Biochem.

[5] Mathews CK (2015). Deoxyribonucleotide metabolism, mutagenesis and cancer. Nat Rev Cancer.

[6] Goldstone DC, Ennis-Adeniran V, Hedden JJ, Groom HC, Rice GI, Christodoulou E, Walker PA, Kelly G, Haire LF, Yap MW, de Carvalho LP, Stoye JP, Crow YJ (2011). HIV-1 restriction factor SAMHD1 is a deoxynucleoside triphosphate triphosphohydrolase. Nature.

[7] Powell RD, Holland PJ, Hollis T, Perrino FW (2011). Aicardi-Goutieres syndrome gene and HIV-1 restriction factor SAMHD1 is a dGTP-regulated deoxynucleotide triphosphohydrolase. J Biol Chem.

[8] Li N, Zhang W, Cao X (2000). Identification of human homologue of mouse IFN-gamma induced protein from human dendritic cells. Immunol Lett.

[9] Laguette N, Sobhian B, Casartelli N, Ringeard M, Chable-Bessia C, Ségéral E, Yatim A, Emiliani S, Schwartz O, Benkirane M (2011). SAMHD1 is the dendritic- and myeloid-cell-specific HIV-1 restriction factor counteracted by Vpx. Nature.

[10] Hrecka K, Hao C, Gierszewska M, Swanson SK, Kesik-Brodacka M, Srivastava S, Florens L, Washburn MP, Skowronski J (2011). Vpx relieves inhibition of HIV-1 infection of macrophages mediated by the SAMHD1 protein. Nature.

[11] Franzolin E, Pontarin G, Rampazzo C, Miazzi C, Ferraro P, Palumbo E, Reichard P, Bianchi V (2013). The deoxynucleotide triphosphohydrolase SAMHD1 is a major regulator of DNA precursor pools in mammalian cells. Proc Natl Acad Sci U S A.

[12] Bonifati S, Daly MB, St Gelais C, Kim SH, Hollenbaugh JA, Shepard C, Kennedy EM, Kim DH, Schinazi RF, Kim B, Wu L (2016). SAMHD1 controls cell cycle status, apoptosis and HIV-1 infection in monocytic THP-1 cells. Virology.

[13] Khoury GA, Baliban RC, Floudas CA (2011). Proteome-wide post-translational modification statistics: frequency analysis and curation of the swiss-prot database. Sci Rep.

[14] Cribier A, Descours B, Valadão AL, Laguette N, Benkirane M (2013). Phosphorylation of SAMHD1 by cyclin A2/CDK1 regulates its restriction activity toward HIV-1. Cell Rep.

[15] White TE, Brandariz-Nuñez A, Valle-Casuso JC, Amie S, Nguyen LA, Kim B, Tuzova M, Diaz-Griffero F (2013). The retroviral restriction ability of SAMHD1, but not its deoxynucleotide triphosphohydrolase activity, is regulated by phosphorylation. Cell Host Microbe.

[16] Drazic A, Myklebust LM, Ree R, Arnesen T (2016). The world of protein acetylation. Biochim Biophys Acta.

[17] Choudhary C, Kumar C, Gnad F, Nielsen ML, Rehman M, Walther TC, Olsen JV, Mann M (2009). Lysine acetylation targets protein complexes and co-regulates major cellular functions. Science.

[18] Zhao S, Xu W, Jiang W, Yu W, Lin Y, Zhang T, Yao J, Zhou L, Zeng Y, Li H, Li Y, Shi J, An W (2010). Regulation of cellular metabolism by protein lysine acetylation. Science.

[19] Seo JH, Cha JH, Park JH, Jeong CH, Park ZY, Lee HS, Oh SH, Kang JH, Suh SW, Kim KH, Ha JY, Han SH, Kim SH (2010). Arrest defective 1 autoacetylation is a critical step in its ability to stimulate cancer cell proliferation. Cancer Res.

[20] Wang Z, Wang Z, Guo J, Li Y, Bavarva JH, Qian C, Brahimi-Horn MC, Tan D, Liu W (2012). Inactivation of androgen-induced regulator ARD1 inhibits androgen receptor acetylation and prostate tumorigenesis. Proc Natl Acad Sci U S A.

[21] Yoon H, Kim HL, Chun YS, Shin DH, Lee KH, Shin CS, Lee DY, Kim HH, Lee ZH, Ryoo HM, Lee MN, Oh GT, Park JW (2014). NAA10 controls osteoblast differentiation and bone formation as a feedback regulator of Runx2. Nat Commun.

[22] Seo JH, Kim HL, Chun YS, Shin DH, Lee KH, Shin CS, Lee DY, Kim HH, Lee ZH, Ryoo HM, Lee MN, Oh GT, Park JW (2016). ARD1-mediated Hsp70 acetylation balances stress-induced protein refolding and degradation. Nat Commun.

[23] Yu M, Ma M, Huang C, Yang H, Lai J, Yan S, Li L, Xiang M, Tan D (2009). Correlation of expression of human arrest-defective-1 (hARD1) protein with breast cancer. Cancer Invest.

[24] Wang ZH, Gong JL, Yu M, Yang H, Lai JH, Ma MX, Wu H, Li L, Tan DY (2011). Up-regulation of human arrest-defective 1 protein is correlated with metastatic phenotype and poor prognosis in breast cancer. Asian Pac J Cancer Prev.

[25] Xu H, Jiang B, Meng L, Ren T, Zeng Y, Wu J, Qu L, Shou C (2012). N-alpha-acetyltransferase 10 protein inhibits apoptosis through RelA/p65-regulated MCL1 expression. Carcinogenesis.

[26] Shim JH, Jiang B, Meng L, Ren T, Zeng Y, Wu J, Qu L, Shou C (2012). Clinical implications of arrest-defective protein 1 expression in hepatocellular carcinoma: a novel predictor of microvascular invasion. Dig Dis.

[27] Lahouassa H, Daddacha W, Hofmann H, Ayinde D, Logue EC, Dragin L, Bloch N, Maudet C, Bertrand M, Gramberg T, Pancino G, Priet S, Canard B (2012). SAMHD1 restricts the replication of human immunodeficiency virus type 1 by depleting the intracellular pool of deoxynucleoside triphosphates. Nat Immunol.

[28] Baldauf HM, Daddacha W, Hofmann H, Ayinde D, Logue EC, Dragin L, Bloch N, Maudet C, Bertrand M, Gramberg T, Pancino G, Priet S, Canard B (2012). SAMHD1 restricts HIV-1 infection in resting CD4(+) T cells. Nat Med.

[29] Descours B, Cribier A, Chable-Bessia C, Ayinde D, Rice G, Crow Y, Yatim A, Schwartz O, Laguette N, Benkirane M (2012). SAMHD1 restricts HIV-1 reverse transcription in quiescent CD4(+) T-cells. Retrovirology.

[30] Mlcochova P, Sutherland KA, Watters SA, Bertoli C, de Bruin RA, Rehwinkel J, Neil SJ, Lenzi GM, Kim B, Khwaja A, Gage MC, Georgiou C, Chittka A (2017). A G1-like state allows HIV-1 to bypass SAMHD1 restriction in macrophages. EMBO J.

[31] Jeong JW, Bae MK, Ahn MY, Kim SH, Sohn TK, Bae MH, Yoo MA, Song EJ, Lee KJ, Kim KW (2002). Regulation and destabilization of HIF-1alpha by ARD1-mediated acetylation. Cell.

[32] Park JH, Seo JH, Wee HJ, Vo TT, Lee EJ, Choi H, Cha JH, Ahn BJ, Shin MW, Bae SJ, Kim KW (2014). Nuclear translocation of hARD1 contributes to proper cell cycle progression. PLoS One.

