# Supplementary material for: SAMHD1 acetylation enhances its deoxynucleotide triphosphohydrolase activity and promotes cancer cell proliferation
Source: Oncotarget. 2017 Jul 31;8(40):68517–29. doi: 10.18632/oncotarget.19704 (PMC5620274; doi:10.18632/oncotarget.19704)
Supplement: Supplementary file 1 [file oncotarget-08-68517-s001.pdf]

SAMHD1 acetylation enhances its deoxynucleotide triphosphohydrolase activity and promotes cancer cell proliferation

SUPPLEMENTARY MATERIALS

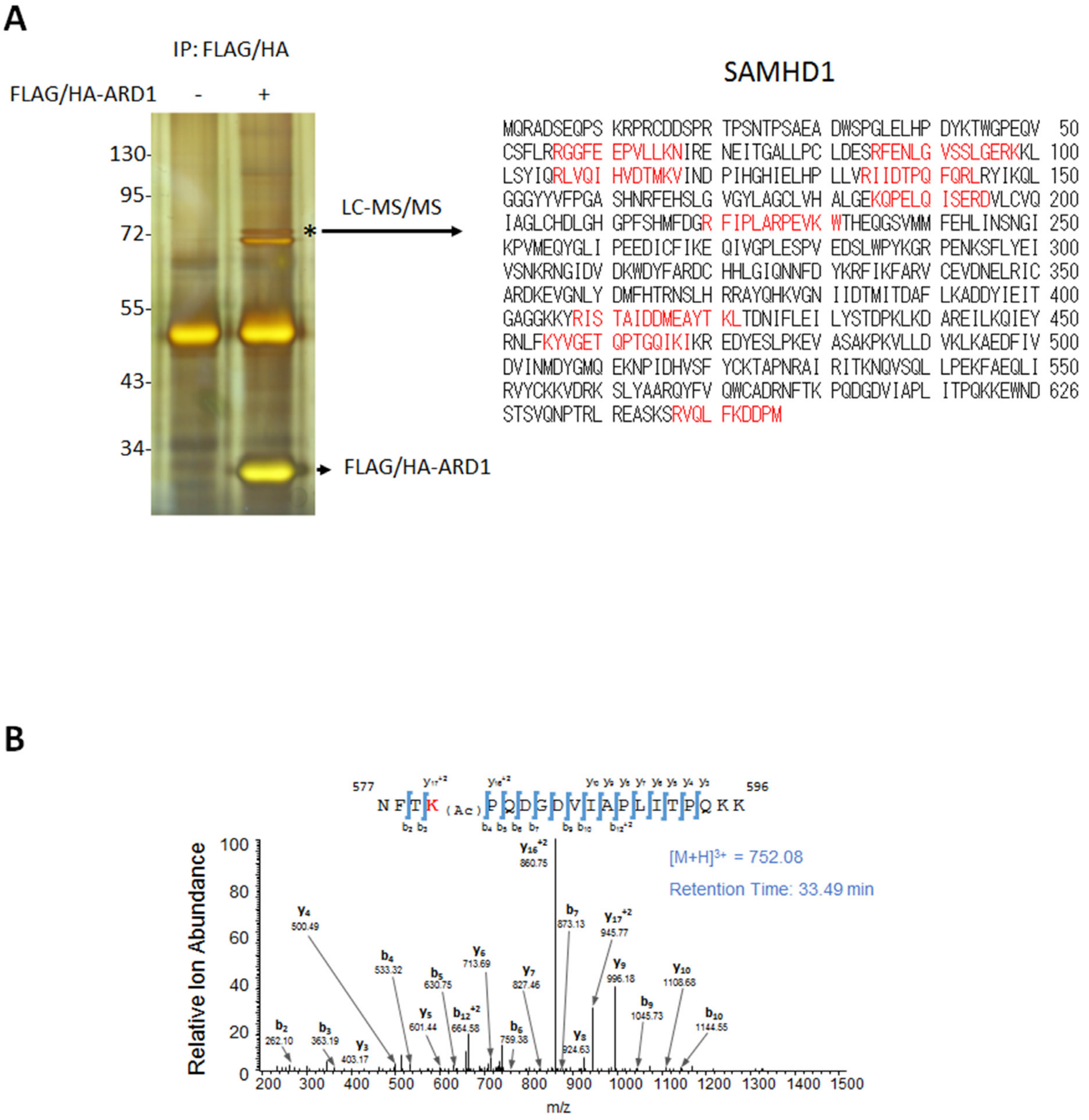

Supplementary Figure 1: ARD1 wildtype binds and acetylates SAMHD1. (A) HEK293T cells were transfected with FLAG/HA-ARD1 and lysed proteins were immunoprecipitated, separated by SDS-PAGE and silver stained. * refers to SAMHD1. **(B)** The acetylation site in acetylated GST-CTD was identified by LC-MS/MS.

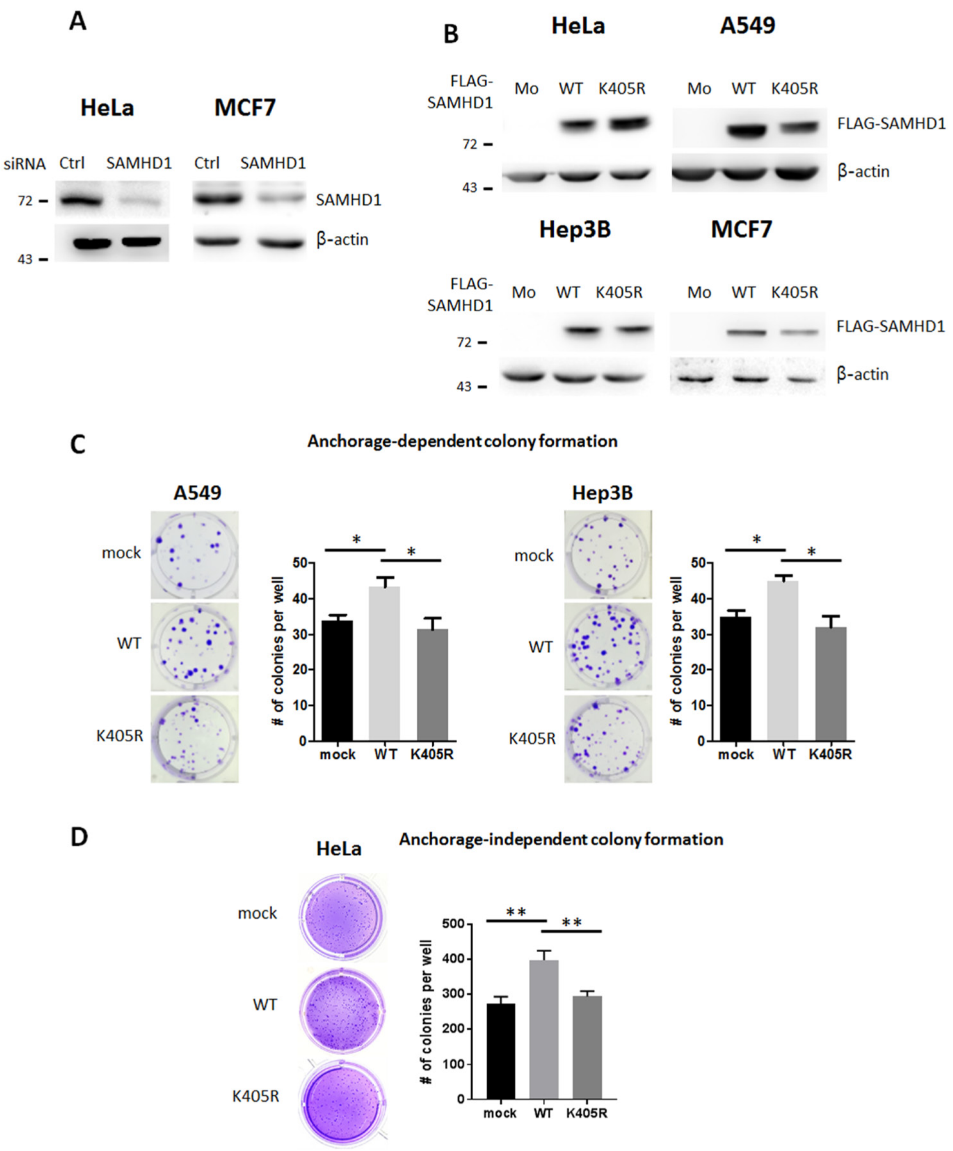

Supplementary Figure 2: SAMHD1 was silenced or overexpressed in various cancer cells. (A) HeLa and MCF7 cells were transfected with siCtrl or siSAMHD1 and were lysed after 48~72h. The level of proteins were assessed by western blot. **(B)** SAMHD1 wildtype or K405R expressing stable cell lines were constructed in HeLa, A549, Hep3B, and MCF7 cells and their protein levels were assessed by western blot. **(C)** Stable A549 and Hep3B cells expressing SAMHD1 wildtype, K405R or empty vector were subjected to anchorage-dependent colony formation assays. Number of colonies were presented as mean ±S.D. (n=3) with representative well pictures. **(D)** Stable HeLa cells expressing SAMHD1 wildtype, K405R or empty vector were subjected to anchorage-independent colony formation assays. Number of colonies were presented as mean ±S.D. (n=3) with representative well pictures.

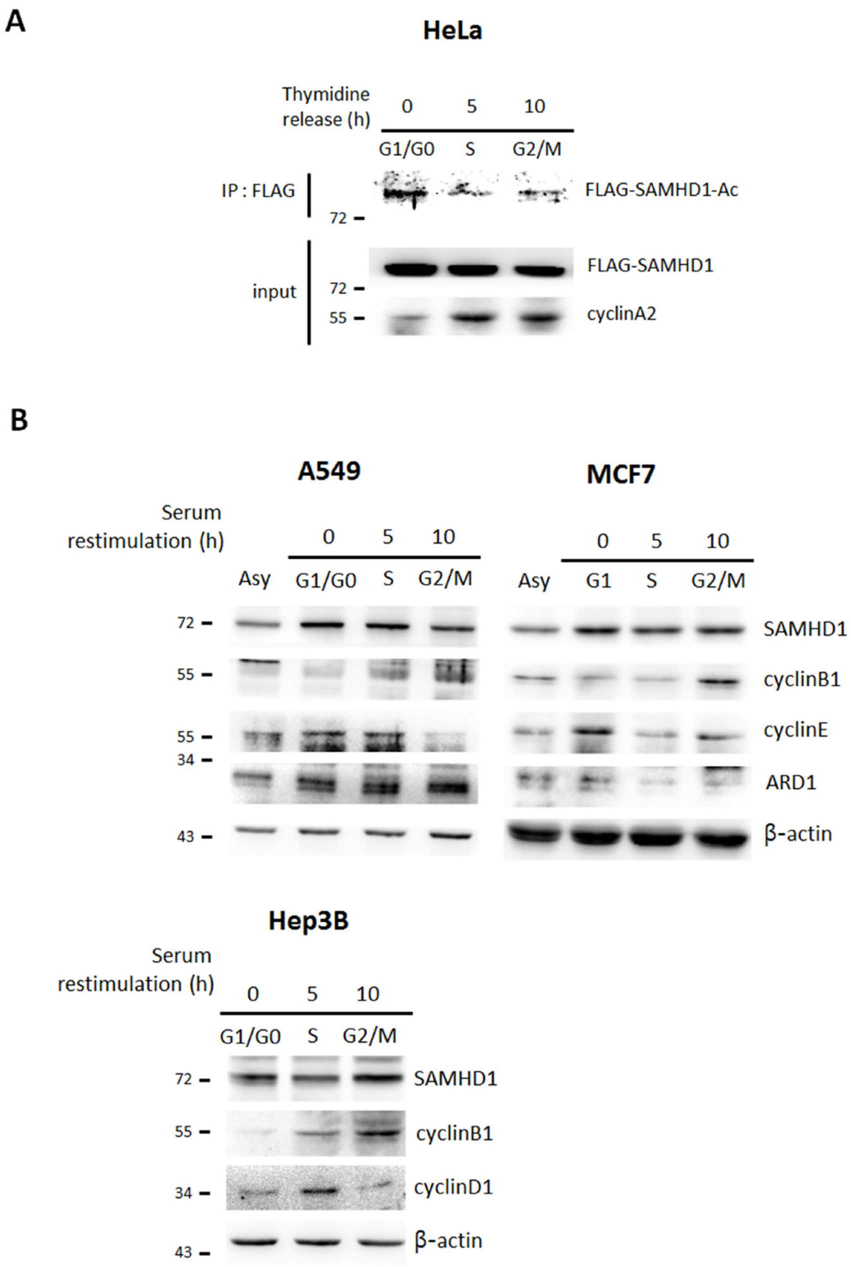

Supplementary Figure 3: SAMHD1 acetylation and expression levels in cancer cells. (A) FLAG-SAMHD1 wildtype expressing stable HeLa cells were synchronized to specific cell cycle phases using thymidine-double block method. SAMHD1 was then immunoprecipitated and blotted with an anti-Lyc-Ac antibody. **(B)** A549, MCF7 and Hep3B cells were synchronized to G1/G0 phase by serum starvation then released for indicated hours to each cell cycle phases. Lysed proteins were assessed by western blot. Asy; asynchornized.

Supplementary Table 1: Clinicopathological characteristics of 7 hepatocarcinoma patients

Patient No.	Age	Gender	Etiology	Tumor size, max (cm)	Procedure	Edmonson grade	Cirrhosis
1	42	M	HBV	1.8	Surgical resection	3	No
2	74	M	NBNC	10.5	Surgical resection	4	No
3	48	M	HBV	3.5	Surgical resection	2	Yes
4	43	M	HBV	3.8	Surgical resection	4	No
5	45	M	HCV	2.5	Surgical resection	3	No
6	70	F	HBV	5.3	Surgical resection	3	Yes
7	56	M	HBV	2.2	Surgical resection	3	No

M, male; F, female; HBV, hepatitis B virus; NBNC, non-B non-C; HCV, hepatitis C virus.
